# Correlates of Post-traumatic Growth Following a Myocardial Infarction: A Systematic Review

**DOI:** 10.1007/s10880-020-09727-3

**Published:** 2020-06-03

**Authors:** Gayle Hegarty, Lesley Storey, Martin Dempster, Dave Rogers

**Affiliations:** 1 School of Psychology, Queen’s University Belfast, University Road, Belfast, UK; 2 Birmingham City University, 4 Cardigan Street, Birmingham, B4 7BD UK; 3 School of Psychology, Centre for Evidence and Social Innovation, Queen’s University Belfast, University Road, Belfast, UK

**Keywords:** Posttraumatic growth, PTG, Myocardial infarction, MI

## Abstract

Correlates of post-traumatic growth (PTG) have been examined in the area of health psychology previously, with much focus on aspects of personality, coping, and social support. This systematic review aimed to examine correlates of PTG for those who have experienced a myocardial infarction (MI). Studies which met inclusion criteria were assessed for quality and reviewed. Results showed an inconsistent strength of associations between studies and so conclusions cannot be drawn. Possible reasons for these differences are discussed and recommendations for future research are suggested.

Ischaemic heart disease is the leading cause of global deaths (World Health Organization, 2018) and myocardial infarction (MI) is the most frequent manifestation of this disease. MI is a sudden and life-threatening event that can be experienced as traumatic (Allan & Sheidt, 1998). Indeed, survivors of MI have been shown to meet diagnostic criteria for Post-Traumatic Stress Disorder (Spindler & Pederson, 2005). A qualitative study of MI found that patients focussed on experiences of pain, fears for the future, the loss of control, and fear of death (López-Medina, Gil-García, Sánchez-Criado, & Pancorbo-Hidalgo, 2016). Additionally, survivors of MI may experience depression or anxiety in response to their diagnosis, the uncertainty surrounding treatment, and concern regarding further attacks (Eriksson, Asplund, Hochwalder, & Svedlund, 2013).

Post-traumatic growth (PTG) is the positive change experienced by individuals that emerges from the struggle following a life challenge or trauma that has impacted an individual’s assumptive world or core beliefs (Tedeschi & Calhoun, 1996). It refers to an enhancement or improvement in psychosocial functioning compared to the individual state before the key event. Initially, coined as ‘perceived benefits’ (Colhoun & Tedeschi, 1990), the term PTG is now used to communicate the transformative experience which is reported by the individual and observed by those close to them (Blackie, Jayawickreme, Helzer, Forgeard, & Roepke, 2015). The concept of PTG has emerged primarily from the study of trauma and how individuals adjust and cope with the same. Tedeschi, Shakespeare-Finch, Taku, & Colhoun (2018) assert this as a constructivist perspective, in that individuals experiencing life challenges will make individual versions of core beliefs and form assumptions based on this to inform their view of themselves, the world and their future. Janoff-Bulman (2004) describes this process of adjustment as finding strength through trauma, psychological preparedness and existential re-evaluation through a search for meaning.

PTG has been identified across a range of health complaints, including serious medical conditions. A systematic review of qualitative studies that examined PTG in life-threatening physical conditions (Hefferon, Grealy, & Mutrie, 2009) found overarching themes of reappraisal of life and priorities, trauma equalling development of self, existential evaluation and new awareness of the body. A systematic review of quantitative studies (Barskova & Oesterreich, 2009) found PTG in those who have a life-threatening disease across 68 studies covering HIV/Aids, cardiac disease, rheumatoid arthritis, multiple sclerosis, spinal cord injuries, orthopaedic injury, burns, lupus and also general disease (mixed study). Additionally, a study by Cordova et al. (2001) compared two groups of age- and education-matched women. One group had been diagnosed with breast cancer, whilst the other was ‘healthy’. They found that whilst both groups had similar levels of well-being and distress, the women with breast cancer displayed PTG. Additionally, Powell, Elkin-Wood, & Collin (2007) conducted a study which found PTG in survivors of brain injury (stroke and traumatic brain injury).

More specifically, PTG has been shown to occur in individuals who have experienced MI, with up to 65% of individuals reporting positive changes (Petrie, Buick, Weinman, & Booth, 1999; Norekval et al., 2008). An influential study by Affleck, Tennen, Croog, & Levine (1987) set out to examine predictors of reoccurrence of MI. This was a longitudinal study, interviewing 247 men at two time points: 7 weeks and 8 years after a MI. Independent of physician’s ratings of prognosis and sociodemographic levels, those who perceived benefits following an MI were less likely to experience a further MI.

There is evidence, therefore, that PTG occurs for some people after experiencing MI and that this can have beneficial outcomes. This leads to the question why does this occur for some people, that is, what factors might explain individual variations in the experience of PTG? Helgeson (2003) measured cognitive adaptation to cardiac events and discovered that higher adaptation predicted lower numbers of cardiac events. Findings such as these lead us to consider which particular aspects of adaptation are associated with PTG and particularly in relation to MI?

Research has focussed on five areas: personality characteristics, cognitive processing, coping mechanisms, social support variables, and mental health, which, of course, may have moderating and mediating effects on each other. Wider PTG research on personality has shown that extraversion correlated positively with PTG, whilst neuroticism had no relationship (Tedeschi & Colhoun, 1996). In terms of how cognitive processing relates to PTG in health conditions, research suggests that perceptions relating to the events are important. For example, perceived intensity of disease (Bellizzi & Blank, 2006) was positively associated with PTG in patients being treated for breast cancer and in another study PTG was found to be positively associated with perceived life threat, whilst it was unrelated to distress or well-being (Cordova et al., 2001). There is some evidence that social support is an important aspect of PTG with the aforementioned systematic review (Barskova & Oesterreich, 2009) finding a positive correlation. This has since been supported by further research (Nenova, DuHamel, Zemon, Rini, & Redd, 2013; Sim, Lee, Kim, & Kim, 2015). In addition, the review found coping strategies to be positively associated with PTG. Finally, consideration of how anxiety or depression might interact with growth is crucial. A systematic review of PTG in cancer patients (Casellas-Grau, Ochoa, & Ruini, 2017) examining psychological and clinical correlates of PTG in cancer found that PTG was inversely associated with anxiety and depression symptoms.

## Rationale and Aims of the Review

The review aims to explore the correlates of PTG in individuals who have experienced MI. The use of psychological theory, knowledge and interventions within the physical health fields continues to grow and given the evidence of PTG in this area, it remains important for psychologists to know what predicates this growth. Not only is this important for intervening with the appropriate person to foster this growth, but it may be utilised in rehabilitation service design and planning. Therefore, the specific review question is what are the factors associated with PTG among people who have experienced a MI and what is the strength of the association between these factors and PTG?

## Method

The review methods followed the PRISMA statement (Moher, Liberati, Tetzlaff, & Altman, 2009).

### Inclusion and Exclusion Criteria

Studies were included if they examined correlates of PTG in individuals who had a diagnosed MI. Intervention studies were excluded due to the possible confounding impact on PTG and the associated variables. Studies which utilized a valid measure of PTG such as (but not confined to) the PTG Inventory (Tedeschi & Calhoun, 1996), Benefit Finding Scale (Tomich & Helgeson, 2002) and the Personal Growth Scale (Garnefski, Kraaij & Spindhoven, 2001) were accepted and correlates had to be examined using a valid psychometric tool. Studies not in English were excluded. Note that criteria were not set in relation to date of study, geographical location, demographics or research setting.

### Search Strategy

Following initial scoping exercises to identify papers relating to predictors of PTG that included related terms such as STEMI and NSTEMI within the specific MI population, a search protocol was developed.

The protocol was registered with PROSPERO (International prospective register of systematic reviews: National Institute for Health Research) in November 2018 (reference PROSPERO 2018 CRD42018114482), with the search taking place in January 2019.

Given that the review straddled medical, psychological and trauma research fields, suitable databases were identified. These were Web of Science, MEDLINE, Scopus, PILOTS and PsycINFO. The databases were searched using key terms (exploded and mapped to subject headings where appropriate) posttraumatic growth OR post-traumatic growth OR post-traumatic growth OR benefit finding OR stress related growth AND myocardial infarction OR MI. The papers were saved in ‘RefWorks’ data management system and duplicates were removed. These papers’ titles and abstracts were then screened by GH and assigned into ‘keep’ or ‘discard’ categories and the former papers were appropriate for full text screening. GH and EM completed this step independently and discussed findings. In addition, a hand search of the references of each of the final papers was conducted to identify any further papers which met the inclusion criteria. No further papers were found.

The tool requires the researcher to rate the papers according to the guidance and their own skilled judgement. Nine of the questions were relevant for these cross-sectional studies (see Appendix Table 3) and so only these nine questions were used for quality assessment and other questions were discarded. Questions deemed not applicable concerned repeated measures, differing levels of exposure and follow-up rates, which are all more suited to longitudinal studies rather than cross-sectional studies. Both GH and DR conducted their assessment independently.

## Results

**Fig. 1:**
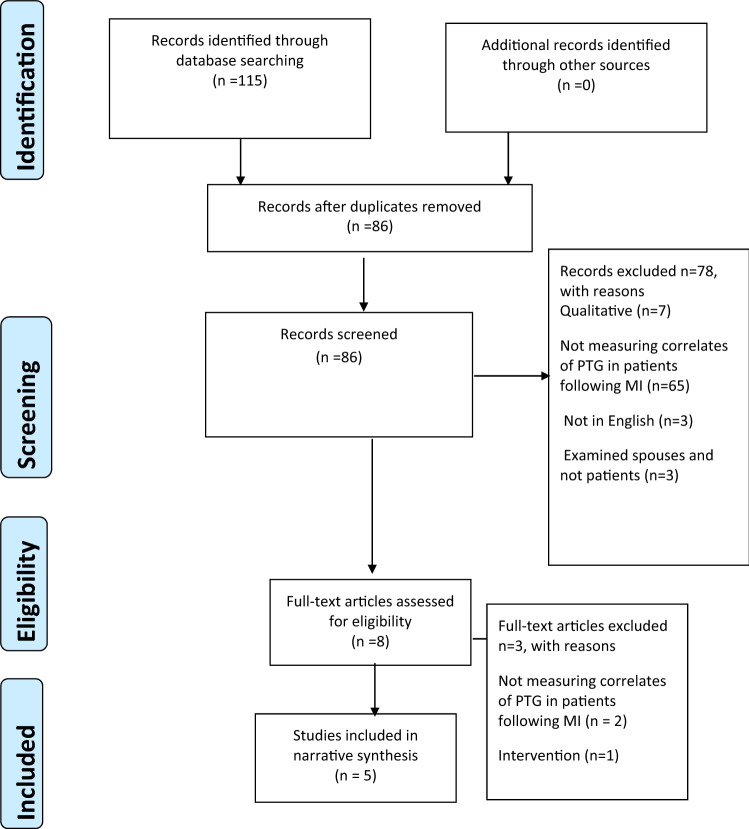
Prisma flowchart

As evidenced in Fig. 1, 115 papers were initially identified by database searches and no further papers were identified from hand searches. Removal of duplicates reduced the papers to 86 and following screening of titles and abstracts eight papers were identified for full text evaluation. Figure 1 identifies the reasons why papers were rejected during the searches at these two points. Five papers met the inclusion criteria with full agreement amongst reviewers.

Methodological Quality of papers: Quality assessment rated two papers ‘good’, two ‘fair’ and one ‘poor’ (see Appendix Table 3). Both reviewers discussed all ratings and reached complete agreement. The studies scored well in terms of objectives, clearly defined populations, independent variables and outcome measures of PTG (dependent variable). One study (Łosiak & Nikiel, 2014) states that the participants were taking part in cardiac rehabilitation, but does not indicate the setting (hospital or community). However, it was considered that the population was likely to be similar in terms of referral to the rehabilitation group. There were variations in recruitment and time since MI in the study by Senol-Durak & Ayvasik (2010). Clarification was requested by email regarding this, but no response was received.

The data extraction table (see Table 1) summarizes the main elements such as study aim, design and measures. Most studies were carried out within hospital populations, using service databases to contact individuals diagnosed with MI (as discussed, with exception Łosiak & Nikiel (2014) which does not clearly state).

**Table 1:** Descriptive information for final sample of studies

References	Sample details	Inclusion & exclusion criteria	Design	PTG measure	Predictor/correlate measure
Study 1: Garnefski et al. (2008)	Netherlands, one medical center, *n* = 139 (246 eligible from database), outpatients in cardiology clinic, male and female, range 35–70 years, information and consent via phone	Inclusion criteria: Over age 30 and under age 70 years, first time MI, length of time from MI 3–12 months, all had intervention of PCI (3–12 months also)	Cross-sectional	Personal Growth Scale adapted elements of PTGI 5 questions, 1–5 Likert scale	Personality (NEO-FFI), mental well-being (HADS, WHO-5) cognitive coping (CERQ)
Study 2: Javed & Dawood (2016)	Pakistan, 4 different hospitals, *n* = 90, male & female. 45–65 years, first MI, Purposive sampling	Inclusion criteria: 45–65 years, 1–36 months since MI. First MI. Exclusion Criteria: No hope for recovery, major surgery after MI, other physical illnesses (except diabetes and hypertension), diagnosis of CVD before MI and diagnosed psychiatric illnesses at point of data collection	Cross-sectional	PTGI	Personality traits (BFI), perceived social support (MSPSS), coping strategies (Brief COPE) Urdu versions of all
Study 3: Łosiak & Nikiel (2014)	Poland, myocardial infarction patients undergoing cardiological rehabilitation following MI, setting not stated, *n* = 53, range 39–68 years, male and female, first MI, 1.5–19 weeks prior	Not stated	Cross-sectional	PTGI (polish version with four factors)	Cognitive coping (CERQ), Experience of life threat (own devised scale)
Study 4: Rahimi et al. (2016)	Iran, 1 hospital’s cardiac clinic, n = 166 (out of 188 eligible), range 21–90 years, male & female, 3–12 months since MI	Inclusion criteria: Min age 21, definite diagnosis of MI, consent, power to communicate, at least 3 months since attack. Exclusion criteria: Severe psychological disorders or Alzheimer’s	Cross-sectional	PTGI	Social Support (CSSS)
Study 5: Senol-Durak & Ayvasik (2010)	Turkey, 4 state hospitals, *n* = 148, (188 asked and 36 declined), 4 excluded after data collection), range 27–80 years, male and female, mini telephone interview and then face to face. Between 1 and 4 MI events, range 2–25,920 days since diagnosis	Excluded if had other life-threatening illness in self, spouse, or child	Cross-sectional	PTGI	Perceived social support (MSPSS), Perception of Event (own devised scale with 2 questions and likert scale) and coping (WCQ with 8 more items added to Turkish version)

*PCI* percutaneous coronary intervention, CVD Cardiovascular disease, *PTGI* Posttraumatic Growth Inventory, *NEO-FFI* Neuroticism Extraversion Openness-Five Factor Inventory, *HADS* Hospital Anxiety & Depression Scale, *WHO-5* Wellbeing Questionnaire, *CERQ* Cognitive Emotion Regulation Questionnaire, *BFI* Big Five Inventory, *MSPSS* Multidimensional Scale of Perceived Social Support, *CERQ* Cognitive Emotion Regulation Questionnaire, *CSSS* Clinical Social Support Scale, *WCQ* Ways of Coping Questionnaire

Not all studies stated explicitly that the participants had a confirmed diagnosis of MI; however, all of the studies detailed the number of MI’s experienced by the participants, and therefore, this was accepted as evidence of MI having occurred.

### Post-traumatic Growth Measure

In all cases, the Post-traumatic Growth Inventory (PTGI; Tedeschi & Calhoun, 1996), or an adapted version, was used to measure post-traumatic growth. The PTGI (Tedeschi & Colhoun, 1996) is an instrument developed to assess positive changes following a traumatic event. The 21-item self-report measure uses a six-point Likert scale (0–5). Five factors are encompassed within the scale, which are denoted in brackets here with corresponding questions. They include ‘I changed my priorities about what is important in life’ (Appreciation of Life), I developed new interests (New Possibilities), I have a greater feeling of self-reliance’ (Personal Strength), ‘I have a better understanding of spiritual matters’ (Spiritual Change), and ‘I have a greater sense of closeness with others’ (Relating to Others). The Personal Growth Scale (Garnefski, Kraaij, Shroevers, & Somsen, 2008) is an adapted 5-item version of the PTGI. Only two aspects of the scale are included: ‘Appreciation of Life’ and ‘Personal Strength’. Garnefski et al. (2008) points to the Principal Component Analysis confirming the one-dimensional structure of the scale and the reliability in the study was high (0.90).

The correlates of PTG examined in the studies varied across studies, but included coping (in 4 studies), social support (in 3 studies), personality (in 2 studies), and mental well-being (in 1 study).

Table 2 shows correlation coefficients of PTG and the factors examined in the five studies. Results show that the correlation coefficients are often limited to one study or are not consistent across studies, and therefore, findings are inconclusive.

**Table 2:** Correlation coefficient of PTG and factors

Factor	Subscale (instrument)	*r* (*n*)	References
Coping	Problem focused coping (WCQ)	.21 (148)	Senol-Durak & Ayvasik (2010)
	Problem focused coping (Brief COPE)	.90 (90)	Javed & Dawood (2016)
	Emotion focused coping (WCQ)	.34 (148)	Senol-Durak & Ayvasik (2010)
	Active emotional coping (Brief COPE)	.85 (90)	Javed & Dawood (2016)
	Avoidant emotional coping (Brief COPE)	− .83 (90)	Javed & Dawood (2016)
	Indirect coping (WCQ)	− .35 (148)	Senol-Durak & Ayvasik (2010)
	Cognitive coping total (CERQ)	.57 (53)	Łosiak & Nikiel (2014)
	Self-blame (CERQ)	.03 (139)	Garnefski et al. (2008)
	Acceptance (CERQ)	.07 (139)	Garnefski et al. (2008)
	Rumination (CERQ)	.03 (139)	Garnefski et al. (2008)
	Positive refocusing (CERQ)	.22 (139)	Garnefski et al. (2008)
	Planning (CERQ)	.15 (139)	Garnefski et al. (2008)
	Positive reappraisal (CERQ)	.48 (139)	Garnefski et al. (2008)
	Putting into perspective (CERQ)	.22 (139)	Garnefski et al. (2008)
	Catastrophizing (CERQ)	.02 (139)	Garnefski et al. (2008)
	Other-blame (CERQ)	− .09 (139)	Garnefski et al. (2008)
Social Support	Social support total (CSSS)	.36 (166)	Rahimi et al. (2016)
	Emotional dimension (CSSS)	.35 (166)	Rahimi et al. (2016)
	Instrumental dimension (CSSS)	.29 (166)	Rahimi et al. (2016)
	Informational dimension (CSSS)	.22 (166)	Rahimi et al. (2016)
	Perceived social support total (MSPSS)	.91 (90)	Javed & Dawood (2016)
	Family support (MSPSS)	.11 (148)	Senol-Durak & Ayvasik (2010)
	Friend support (MSPSS)	.24 (148)	Senol-Durak & Ayvasik (2010)
	Significant support (MSPSS)	.18 (148)	Senol-Durak & Ayvasik (2010)
Personality	Extraversion (BFI)	.63 (90)	Javed & Dawood (2016)
	Extraversion (NEO-FFI)	.30 (139)	Garnefski et al. (2008)
	Agreeableness (BFI)	.53 (90)	Javed & Dawood (2016)
	Agreeableness (NEO-FFI)	.09 (139)	Garnefski et al. (2008)
	Conscientiousness (BFI)	.73 (90)	Javed & Dawood (2016)
	Conscientiousness (NEO-FFI)	.34 (139)	Garnefski et al. (2008)
	Neuroticism (BFI)	− .81 (90)	Javed & Dawood (2016)
	Neuroticism (NEO-FFI)	− .22 (139)	Garnefski et al. (2008)
	Openness to experience (BFI)	.85 (90)	Javed & Dawood (2016)
	Openness (NEO-FFI)	− .03 (139)	Garnefski et al. (2008)
Mental well-being	Depression (HADS)	− .39 (139)	Garnefski et al. (2008)
	Positive wellbeing (WHO-5)	.38 (139)	Garnefski et al. (2008)

*NEO-FFI* Neuroticism Extraversion Openness-Five Factor Inventory, *HADS* Hospital Anxiety & Depression Scale, *WHO-5* Well-being Questionnaire, *CERQ* Cognitive Emotion Regulation Questionnaire, *BFI* Big Five Inventory, *MSPSS* Multidimensional Scale of Perceived Social Support, *CERQ* Cognitive Emotion Regulation Questionnaire, *CSSS* Clinical Social Support Scale, *WCQ* Ways of Coping Questionnaire

### Coping

Four studies examined the role of coping in PTG (Garnefski et al., 2008; Senol-Durak & Ayvsik, 2010; Łosiak & Nikiel, 2014; Javed & Dawood, 2016). Two studies (Garnefski et al., 2008; Łosiak & Nikiel 2014) used the Cognitive Emotion Regulating Questionnaire (CERQ; Garnefski et al., 2001); however, only Łosiak & Nikiel (2014) found a strong positive relationship to exist. Garnefski et al., (2001) did not report the total CERQ correlation, but rather the subscales. These, overall, found a weak-to-moderate association with PTG.

Two studies examined problem-focussed coping. Javed & Dawood (2016) used the Brief COPE (Carver, 1997) and Senol-Durak & Ayvasik (2010) administered the Ways of Coping Questionnaire (Folkman et al., 1988) and whilst both found a positive association these varied from weak to strong, and therefore, they are not consistent. Indeed, the study conducted by Javed & Dawood (2016) had the strongest association between coping and PTG on all subscales. However, the strength of these findings was not supported by the other three studies.

### Social Support

Three of the studies (Senol-Durak & Ayvasik 2010; Javed & Dawood 2016; Rahimi, Heidarzadeh, & Shoaee, 2016) addressed the area of social support and PTG. Whilst all studies found a positive association between the variables, the strength of this relationship varied, and it was difficult to directly compare the data between studies. Both Javed & Dawood (2016) and Senol-Durak & Ayvasik (2010) examined perceived social support using the same self-report measure (Multidimensional Scale of Perceived Social Support; Zimet, Dahlem, Zimet, & Farley, 1988); however, one reported subscale coefficients and the other presented total scale association. There was a marked difference in the strength of association, with Senol-Durak & Ayvasik (2010) finding a weak positive association and Javed & Dawood (2016) reporting a strong positive relationship between the variables.

### Personality

Two studies examined the relationship between personality and PTG (Garnefski et al., 2008; Javed & Dawood, 2016) and used the Neuroticism Extraversion Openness-Five Factor Inventory (Costa & McCrae, 1992) and Big Five Inventory (John & Srivastava, 1999) scales, respectively, to measure these constructs. Whilst different self-report measures were administered, they both contained the same five subscales of extraversion, agreeableness, conscientiousness, neuroticism, and openness to experience and are, therefore, comparable. Table 2 shows that whilst the study by Javed & Dawood (2016) reported moderate-to-strong associations between the subscales and PTG, Garnefski et al. (2008) found weak to negligible associations mainly. For example, Javed & Dawood (2016) found neuroticism to be strongly negatively associated with PTG (*r* = − .81, *n* = 90), with Garnefski et al. (2008) reporting a weak negative relationship (*r* = − .22, *n* = 139). In fact, Javed & Dawood (2016) reported a strong correlation with all aspects of personality, presenting a strong positive relationship with extraversion, agreeableness, consciousness, and openness to experience and a strong negative relationship with neuroticism. So, despite comparable psychometric measures, results are inconsistent.

### Mental Well-Being

One study examined the relationship of mental well-being and PTG (Garnefski et al., 2008). The measures to determine mental well-being were the Hospital Anxiety and Depression Scale (HADS: Snaith, 2003) and the WHO-5 Well-being Questionnaire (Bech, 1998). A decision to only use the depression subscale from the measure was made without adequate explanation. Therefore, the anxiety subscale was not used. A moderate negative correlation between PTG and depression was found and a moderate positive correlation with well-being. Given that these variables and results feature in only one study, no definitive conclusions can be drawn within the review.

## Discussion

This review aimed to determine the factors associated with PTG among people who have experienced a MI and the strength of the association between these factors and PTG. The five papers that met inclusion criteria examined four factors: coping, social support (and perception of), personality, and psychological well-being. Results showed inconsistent strength of associations across all four variables, so no conclusions regarding the relationship between these variables and PTG can be drawn. It is important to examine why this may be the case, in the context of this health condition in relation to wider health and PTG literature and factors which may be impacting such as type of study design, methodology and study quality.

The role of personality in how individuals experience PTG has already been highlighted. Tedeschi & Colhoun (1996) found that extraversion was most likely to correlate with PTG and neuroticism was not associated with growth. These findings were corroborated by Sheikh (2004) in the context of heart disease patients in the UK and the USA. The study by Garnefski et al. (2008) followed this pattern of findings, but contrary to previous research, another study found extraversion to be strongly positively correlated with PTG, but neuroticism was strongly negatively correlated with PTG (Javed & Dawood, 2016). There were also differences between the studies in the strength of the positive relationship.

These findings could be related to several differences between the studies. The research teams used different PTG and personality measures and there were differences in homogeneity of the study populations. Garnefski et al. (2008) only examined those who had percutaneous coronary intervention (PCI) and limited their participants to 3–12 months after this point. Javed & Dawood (2016) did not address whether participants had a medical procedure such as a PCI and they examined participants 1–36 months from MI diagnosis. The impact of a procedure such as a PCI might influence views on ‘New Possibilities’ or ‘Appreciation of Life’ as measured in the PTGI (Tedeschi & Calhoun, 1996). This in turn could influence coping and perceived support, which in turn could affect growth levels. Therefore, a difference in participant experiences may account for this disparity in association.

Cultural differences across the studies are also conceivable. Each of the five studies were conducted in different countries. Whilst we are aware that PTG as a phenomenon occurs globally (Sawyers, Ayers, & Field, 2010; Weiss & Berger, 2010), studies have also shown differences, such as higher levels of PTG in the USA compared to much lower levels in Japan (Taku, 2010). Researchers have hypothesised that some societies (such as the USA) promote self-enhancement and there is a societal pressure to report growth (Zoellner, Rabe, Karl, & Maercker, 2008).

Tedeschi et al. (2018) are keen to point out differences in individualistic and collective norms within society, which influence how individuals attach meaning to and respond to trauma. Moreover, the studies all used translated version of the original PTGI (Tedeschi & Colhoun, 1996). One study (Garnefski et al., 2008) used an adapted version, whilst another (Łosiak & Nikiel, 2014) found validity in a four-factor version of the scale when translated into Polish. It is unclear how these differences have impacted on associations and the comparisons of such across the five studies.

Following on from this point, it is worth considering differences in health care systems across the studies. None of the studies took place in the United Kingdom, where referral to non-governmental organisations (such as The Chest, Heart and Stroke Association) for rehabilitation is standard practice and it may be protocol for MI survivors in other countries to be referred to health service, community support and rehabilitation groups. None of the studies recorded participant involvement with such support and yet research shows how informational and social support increases PTG (Nenova et al., 2013; Sim et al., 2015).

In addition, the way in which individuals’ access health care may be dissimilar and there may be differences in how health information is communicated either individually by practitioners, or systemically. These aspects, along with individual, societal or cultural norms, are likely to lead to variances in associations which are not easily identified across the studies.

Whilst several of the studies controlled for previous MI events, psychiatric or physical illness and major surgery, none appeared to consider impact of previous trauma. This appears to be a deficit across other research studies, as highlighted by Bostock, Sheikh, & Barton, (2009) in a systematic review of health-related trauma. Given that assumptive core beliefs are an integral part of PTG and the individual’s subsequent adaptation to the event (Tedeschi & Colhoun, 1996), their trauma history will undoubtedly have an impact on their world view. MI is a sudden threat to life, and the theory of PTG incorporates the psychological reaction to this, so the occurrence of more than one of these events will increase the trauma burden for said individuals. It is likely that this would impact on PTG. Whilst recruiting from a more heterogeneous group has ecological validity, results are likely to be affected by these successive traumas.

Following this theme, there were differences in number of MI’s experienced. In one of the studies, individuals had experienced between one and four MI’s. Three of the other studies included only those who had experienced their first MI, whilst Rahimi et al. (2016) did not disclose this statistic. If MI is to be considered a traumatic event, and the literature supports this hypothesis, the number of MI’s experienced should be considered within the research. This may impact on how an individual adjusts to their trauma, their PTG and factors such as psychological well-being and coping.

Study quality should be considered. The quality evaluation tool was designed for cross-sectional studies, and the assessment concluded that two of the studies were good, two were fair and one was weak. This may have contributed to discrepancies in the findings across the studies. For example, a ‘good’ (Javed & Dawood, 2016) and ‘weak’ (Senol-Durak & Ayvasik, 2010) study examined perceived social support using the same scale, but reported strong and weak positive associations, respectively. Whilst the Senol-Durak & Ayvsik (2010) study met inclusion criteria, its methodology lacked rigour, and vital elements such as time from MI or number of MI’s experienced, were not considered.

It is noted that the studies differed in time from when the MI occurred. Whilst two studies (Rahimi et al., 2016; Garnefski et al., 2008) conducted their studies 3 to 12 months after MI, Javed & Dawood’s (2016) study spanned 1 to 36 months. The study by Senol-Durak & Ayvasik (2010) recorded time from MI as being between 2 and 25,920 days (which is over 71 years). Therefore, the question of what was being measured arises. Bostock et al. (2009) in a systematic review of PTG and optimism in health-related trauma points out that time must elapse to allow growth to develop, but the general literature does not stipulate optimum timescales involved. It does appear that there is no defined course of PTG development. For example, Stanton, Bower, & Low (2006) state that PTG appears to be higher in the year or two following an event, but may then diminish. Therefore, time from event should be considered in study design as variations can make it difficult to draw comparisons between studies.

## Limitations and Future Directions

Although this review was robust in its protocol and analysis, there were a few limitations that should be highlighted. The small number of studies identified examined a narrow range of variables which can impact on PTG. For example, distress levels and anxiety (Barskova & Oesterreich, 2009), or illness perceptions (Leung, Gravelly-Witte, Macpherson, & Irvine, 2010) were not included in these studies. The protocol excluded studies that were not in English. These may have added to our review.

Future research should consider confounding variables such as time from diagnosis, number of MIs’ experienced and other interventions (medical and social) that the population may have experienced. Longitudinal research specifically to ascertain PTG levels following MI would be extremely valuable. This could inform timing of interventions. Future studies could also consider examining other factors such as illness perceptions and anxiety. All the studies examined were cross-sectional, and so causation and direction of influence cannot be extrapolated. A study using a longitudinal design would be valuable in examining measures at several time points.

## Conclusions

This is the first systematic review to examine the correlates of PTG in individuals who have experienced an MI and the strength of such associations. The five cross-sectional studies addressed the association between PTG and social support, coping, psychological well-being and personality. The results showed an inconsistent pattern of association; therefore, conclusions cannot be drawn. Possible contributing factors such as study quality, previous trauma, cultural or methodological differences were highlighted. These findings have implications for future research, in that longitudinal studies which take account of these confounding variable are recommended, so that direction of association can be extrapolated.

## Appendix

See Table 3.

**Table 3:** Showing results of quality assessment using quality of observational cohort and cross-sectional studies (NHLBI, 2014)

Author(s)	Was the research question or objective clearly stated?	Was the study population clearly specified and defined?	Was the participation rate of eligible persons at least 50%	Were all the subjects selected or recruited from the same or similar populations (including the same time period)? Were inclusion and exclusion criteria for being in the study prespecified and applied uniformly to all participant?	Was a sample size justification, power description, or variance and effect estimates provided?	Was the timeframe sufficient so that one could reasonably expect to see an association between outcome and exposure if it existed?	Were the exposure measures (independent variables) clearly defined, valid, reliable, and implemented consistently across all study participants?	Were the outcome measures (dependent variables) clearly defined, valid, reliable, and implemented consistently across all study participants?	Were key potential confounding variables measured and adjusted statistically for their impact on the relationship between exposure and outcome?	Quality Assessment of study
Garnefski, Kraaij, Shroevers, & Somsen (2008)	Yes	Yes	Yes	Yes	No	Yes	Yes	No	Yes	Fair
Javed & Dawood (2016)	Yes	Yes	NR	Yes	Yes	Yes	Yes	Yes	Yes	Good
Łosiak & Nikiel (2014)	Yes	Yes	Yes	Yes	No	No	Yes	Yes	Yes	Fair
Rahimi, Heidarzadeh, & Shoaee (2016)	Yes	Yes	Yes	Yes	Yes	Yes	Yes	Yes	No	Good
Senol-Durak & Ayvasik (2010)	Yes	Yes	Yes	Yes	No	No	Yes	Yes	Yes	Weak
